# Differences in the Perception of Dietary Supplements between Dietary Supplement/Medicine Users and Non-Users

**DOI:** 10.3390/nu14194114

**Published:** 2022-10-03

**Authors:** Tsuyoshi Chiba, Nanae Tanemura

**Affiliations:** Department of Food Function and Labeling, National Institute of Health and Nutrition, National Institutes of Biomedical Innovation, Health and Nutrition, Tokyo 162-8363, Japan

**Keywords:** perception, dietary supplements, medicine, anxiety

## Abstract

Some patients use dietary supplements and medicines concomitantly, with an inappropriate perception of their safety and efficacy. To clarify the perception between dietary supplement and medicine users and non-users, we conducted an internet survey. In this survey, 38.9% of participants used dietary supplements, 32.6% used prescription medicines, and 14.7% used dietary supplements and prescription medicines concomitantly. Then, we conducted a further survey on four groups, dietary supplement and prescription medicine users, dietary supplement only users, prescription medicine only users, and non-users (500 each). Dietary supplement users had favorable outcomes in terms of both the safety and efficacy of dietary supplements compared to dietary supplement non-users. This perception of dietary supplements was independent from medicine use. The awareness of the Health Food Network consumer navigation site, which provides information about dietary supplements for consumers, was the highest among dietary supplement and prescription medicine users, but it was still low (2.2%). In conclusion, consumers who use dietary supplement and prescription medicine concomitantly have favorable outcomes for their safety and efficacy and a low awareness of their interaction. There is a need to provide information, especially regarding the risk of interaction, that takes into account the consumer’s situation.

## 1. Introduction

Many people worldwide use dietary supplements [1], and most of them think that dietary supplements, especially natural products, are safe. Previous surveys have shown that approximately 20% of consumers reported concomitantly using dietary supplements and medicines [2]. In addition, the concomitant use of dietary supplements and medicines is reported in not only adults but also adolescents in Japan [3]. Adverse events resulting from dietary supplement use have been reported [4,5,6]. However, attractive health claim leads to consumer purchasing behavior, especially in patients. The health claims of dietary supplements are regulated in each country, and claims for treatment diseases are prohibited in common [7]. So, most dietary supplements implicitly claim to have efficacy in treatment of diseases, and these claims mislead consumers to use dietary supplements for the treatment of diseases [8,9]. In addition, it is reported that many ingredients of dietary supplements, especially botanicals, have the potential to interact with drugs [10,11], and their concomitant use can cause serious adverse events [12]. In this regard, particular attention should be paid to the use of dietary supplements by patients.

Most patients think that dietary supplements are equivalent to food and that they do not interact with their medication. In this regard, healthcare professionals, especially physicians and pharmacists, must consult with their patients about dietary supplement use. However, only 30% of patients inform their doctors or pharmacists of their dietary supplement use because most of them believe that dietary supplements are equivalent to food and that concomitant usage is safe [2]. It is also reported that doctors and pharmacists do not have sufficient knowledge of dietary supplements because education about this topic is insufficient in the university curriculum. Therefore, they do not regularly ask their patients about dietary supplement usage [13,14], and they cannot provide them with appropriate consultation [15,16]. In addition, only 18% of dietary supplement users declared dietary supplement use to their clinicians, and only 20% of dietary supplement users were asked about their dietary supplement use upon admission by their clinicians [17].

Since December 2019, the novel coronavirus disease (COVID-19) has altered lifestyles worldwide. Vaccination is continuously being carried out, but the efficacy of vaccines against the mutated severe acute respiratory syndrome coronavirus 2 (SARS-CoV-2) virus may be low, and the number of infected people is increasing. Indeed, as of August 2022, Japan has the highest number of infected people in the world, even though more than 80% of people have had their second vaccination and more than 60% have had their third vaccination. After the spread of COVID-19, many dietary supplements on the market claim to have anti-COVID-19 effects without supporting scientific evidence. A total of 8.3% of Japanese people use dietary supplements for the prevention of COVID-19 [18], and this usage is even higher in other countries [19]. Nirmatrelvir, a COVID-19 medicine, is metabolized by CYP3A4. Cannabidiol (CBD), one of the cannabinoids derived from hemp (*Cannabis sativa*), also became popular during the COVID-19 pandemic, not only in Japan, but across the world because CBD is a potential alternative treatment for anxiety, depression, and psychotic disorders [20]. In addition, CBD has inhibitory effects against SARS-CoV-2 infection [21] and replication [22]. However, CBD can inhibit CYP3A4 activity [23], indicating that the concomitant use of CBD and Nirmatrelvir has a risk of interaction.

As patients taking prescription medicines should be aware of the safety of dietary supplement usage, we investigated whether there were differences in the perception of patients related to dietary supplements and depending on their medication status.

## 2. Materials and Methods

### 2.1. Participants and Procedures

A cross-sectional online questionnaire survey was conducted by Rakuten Insight, Inc. (Tokyo, Japan) between 10 and 15 December 2021. The questionnaire consisted of a preliminary survey and a further survey. The preliminary survey was conducted on 10,129 respondents aged 20–79 years old. Exclusion criteria were employment in jobs associated with medical and nutritional services and experience in the developmental research of dietary supplements. The preliminary survey was conducted to elucidate dietary supplement usage and prescription medication status. Then, a further survey was conducted on 2000 respondents from the preliminary survey on the basis of residence, sex, and age. Four groups were defined: Group 1: dietary supplement and prescription medicine users (Both), Group 2: dietary supplement users (DS), Group 3: prescription medicine users (PM), and Group 4: non-users of both dietary supplement and prescription medicine (None). The further survey consisted of the following questions about participants’ attitudes towards dietary supplement advertisements in the hypothetical setting, the perception of dietary supplements, and the awareness and usage of the Health Food Network (HFNet) site for consumer navigation, the website produced by the National Institutes of Biomedical Innovation, Health and Nutrition. This study was conducted in accordance with the Declaration of Helsinki and was approved by the Research Ethics Committee of the National Institutes of Biomedical Innovation, Health and Nutrition (no. 329, 10 November 2021). The questionnaire in this survey is presented as a Supplementary File S1. The detailed procedures of the internet survey are described in a previous report [24].

### 2.2. Statistical Analysis

Anxiety towards present and future health was assessed on 5-point Likert-type scales (1 = not at all to 5 = extremely). The statistical difference in the mean score was determined using one-way ANOVA with Dunnett’s post hoc tests. Differences in distribution among groups were compared using the chi-squared (χ^2^) test. All statistical analyses were performed using IBM SPSS Statistics ver. 28.0.1.0 (IBM Corporation, Armonk, NY, USA). A *p*-value of <0.05 was considered statistically significant.

## 3. Results

### 3.1. Characteristics in the Preliminary Survey

The 10,129 respondents (43.5 ± 13.9 years old) comprised 4744 males (42.9 ± 15.0 years old) and 5385 females (44.1 ± 12.8 years old) (Table 1). The results show that 32.6% of participants were taking medicines (including OTC: 40.1%), and 38.9% of participants were using dietary supplements (Table 2). Among them, 14.7% of participants were concomitantly using dietary supplements and prescription medicines, and 4.7% of participants were concomitantly using dietary supplements and OTC medicines (Table 3).

### 3.2. Self-Care Practice

We asked participants about the types of self-care practices they conduct (Table 4). The most popular self-care practice was “regular health check”, conducted by 61.8%, with 21.7% intending to do so in the future, followed by “vaccination”, “securing sleep time”, and “rest/refresh”. In this survey, “dietary supplement use” was seventh, with 36.8% of participants and 16.3% intending to do so. However, the prevalence of “I conducted it in the past, but I do not do it now” was the highest in “dietary supplement use” (21.6%). In addition, consultations with doctors (29.1%), pharmacists (46.0%), and dietitians (60.8%) were unpopular in self-care practice.

### 3.3. Characteristics in the Further Survey

Then, we conducted a further survey on a total of 2000 participants, i.e., 500 dietary supplement and prescription medicine users (Both), 500 dietary supplement users (DS), 500 prescription medicine users (PM), and 500 non-users (None), using age, gender, and area of residence as allocation factors (Table 5). Most participants in Both and PM were diagnosed with some kind of disease, such as hypertension, dyslipidemia, diabetes mellitus, or cancer. In addition, some patients with these diseases were even found in DS and None, and a proportion of them used OTC medicines for treatment of these diseases.

### 3.4. Anxiety

First, we asked participants about anxiety towards present and feature health (1 = not at all to 5 = extremely anxious) (Table 6). For anxiety towards present health, the mean score was 3.42 in Both, 3.33 in PM, 2.87 in DS, and 2.72 in None. This indicates that the anxiety was higher in participants taking medicines, but there was no difference between supplement users and non-users. On the other hand, for anxiety towards future health, the mean score was 3.74 in Both, 3.61 in PM, 3.39 in DS, and 3.06 in None, indicating that the overall level of anxiety towards future health was higher than that for present health. There was no difference between Both and PM, but the difference between DS and None caused the greatest increase in the level of anxiety in the DS group (0.52 points) compared to other groups (Both: 0.32, PM: 0.27, None: 0.34) from Present to Future.

### 3.5. Attitude towards Dietary Supplement Advertisements in the hypothetical Setting

We asked participants the following question in a hypothetical setting, “Your doctor advised that you might have XX (some kind of disease) and you should take medicine for it. On your way home, you saw an advertisement in the train, and it stated that ‘This dietary supplement improves XX’. What would you do?” In Both, half of them answered that they would use medicine only, and 40.8% of them answered that they would use both (Table 7). In PM, two-thirds of them answered that they would only use medicines, and only 11.2% of them answered that they would use both. On the other hand, in DS, 42.6% of them answered medicine only, and 36.0% of them answered both. In None, 42.2% of them answered medicine only, and 40.0% of them answered that they would not use both. Even though the prevalence of dietary supplement only was low (1.4–5.8%) in all groups, it was the highest in DS.

### 3.6. The Perception of Dietary Supplements

In order to identify differences in the perception of dietary supplements according to the situation of dietary supplement and medicine use, 16 questions about the perception of dietary supplements were asked, and answers ranged from “strongly disagree” to “strongly agree” (Table 8). Compared to dietary supplement non-users (PM and None), and more dietary supplement users (Both and DS) had a safe perception, with answers such as “It is safe because it is food”, “It is safe because it is made from natural ingredients or herbs”, “It has fewer side effects than pharmaceuticals”. They also had effective perception, with answers such as “It can be effective”, “It can prevent diseases”, and “It can treat diseases”. Furthermore, more dietary supplement users (Both and DS) had a demand to use dietary supplements for “beauty and diet” and “muscle building”. There was no difference between medicine users and non-users (Both vs. DS, PM vs. None).

### 3.7. Awareness of the Health Food Network Consumer Navigation Site

We asked participants about their awareness of the HFNet consumer navigation site provided by the National Institutes of Biomedical Innovation, Health and Nutrition (Table 9). The highest percentages of “I know this site” and “I have seen this site” were in Both (2.2% and 3.8%, respectively). On the other hand, the lowest percentages of “I know this site” and “I have seen this site” were in PM (0.4% and 1.8%, respectively). Then, we asked participants about their utilization of these sites (Table 10). A few participants (from 0.2% in PM to 1.2% of Both) answered “I already use it”, even if they were using dietary supplements. However, 25.2% of Both and 20.8% of DS answered, “I want to use it in the future”. This result indicate that the HFNet consumer navigation site might be useful for dietary supplement users.

## 4. Discussion

In this survey, almost half of the consumers used dietary supplements as a self-care practice, while 14.7% of consumers used dietary supplements and prescription medicines concomitantly. Our previous surveys showed that approximately 70% of those who concomitantly used dietary supplements and medicines did not inform their doctor or pharmacist about their dietary supplement use [2,25]. In this situation, the appropriate perception of dietary supplements in each individual, especially patients, is important. However, the perception of dietary supplements depended on dietary supplement use but was independent from prescription medicine use, and dietary supplement users had a more favorable perception of dietary supplements than non-users. In addition, we found different attitudes among groups in the hypothetical setting. As expected, almost half of Both group would have liked to use medicine only, and the other half of them would have liked to use both dietary supplement and medicine for new diagnoses. Similar results were found in DS. One-third of participants in DS would have used both dietary supplement and prescription medicine when diagnosed with a new disease. This means that participants in DS have the potential to become participants in the Both category in the future.

The hypothetical setting can happen in the real world. A lot of dietary supplements implicitly claim to have efficacy in the treatment of diseases, and patients use these products for the treatment of diseases [2,8,9,25]. In addition to attractive health claims, more dietary supplement users including patients (Both and DS) thought “It is safe because it is food” “It is safe because it is made from natural ingredients or herbs” and “It has fewer side effects than medicines” compared to non-users (PM and None) in this study (Table 8). These perceptions encouraged patients to use dietary supplements. Previously, both medicines and dietary supplements were regulated by the Ministry of Health, Labour and Welfare in Japan. However, the regulation of dietary supplements was relegated to the Consumer Affairs Agency, Government of Japan in 2009, which made it difficult for the Government to uniformly regulate the use of medicines and dietary supplements. In this regard, the role of healthcare professionals in dietary supplement use in patients is important.

Previously, we surveyed pharmacists and dieticians working at pharmacies or hospitals regarding the concomitant usage of dietary supplements and drugs among their patents and found some serious adverse events associated with the concomitant use of dietary supplements and medicines [26]. In this survey, the most popular combination was warfarin and aojiru. Aojiru is a powder or drink made from green leafy vegetables such as young leaves of barley, *Angelica keiskei (Miq.) Koidz*, and *Brassica oleracea L. var. acephala DC*, and it is a popular dietary supplement in Japan. Aojiru may contain high doses of vitamin K and interfere with the anticoagulant activity of warfarin [27,28]. Anti-hypertensive agents and grapefruit juice was another major combination in these patients. However, the diverse contents of flavonoids (e.g., naringin and naringenin) and furanocoumarins (e.g., bergamottin and 6′,7′-dihydroxybergamottin) are reported among grapefruit species [29,30]. This makes it difficult to estimate their interaction with drugs. Finally, St. John’s wort (*Hypericum perforatum L.*) is a popular herbal supplement for depression, not only in Japan, but across the world. However, St. John’s wort is a potent inducer of human CYP3A4 and P-glycoprotein and has pharmacodynamic interactions with some drugs [31,32,33]. The Ministry of Health, Labour and Welfare in Japan has issued a warning to consumers regarding the concomitant use of St. John’s wort and medicines. 

Warfarin is one of the most well-known and sensitive drugs in drug–food interactions because the enhancement of anticoagulant activity by warfarin can induce intracranial bleeding that leads to death. Recently, a systematic review of warfarin was published, including 149 articles and 78 herbs, food, or dietary supplements, such as chamomile tea, cannabis, chitosan, green tea, ginkgo biloba, ginger, and St. John’s wort [34]. In addition, systematic reviews of interactions between dietary supplements and other drugs, such as cardiovascular drugs [35], carbamazepine [36], antiretroviral drugs [37], and levothyroxine [38], have recently been conducted. Cancer patients prefer to use dietary supplements as complementary alternative medicines [39,40,41], and 61.9% (26/42) of cancer patients were using dietary supplements in this study (Table 5). Dietary supplement usage not only causes adverse events but also interacts with treatment for cancer in patients [42,43,44].

Bioactive compounds in dietary supplements interact with drugs via pharmacokinetic and/or pharmacodynamic mechanisms [45]. However, it is reported that physicians and pharmacists do not have enough knowledge of dietary supplements and their possible interactions with drugs [13,14]. Therefore, the perceptions of dietary supplements in patients are important. In terms of “Concomitant use with medicine is safe, because it is food”, 40% of participants who were taking medicines only (PM) answered “Strongly disagree” or “disagree”, suggesting that they may not use dietary supplements because they are careful about using them in combination with other drugs. However, the perception of the concomitant use of dietary supplement and medicine was almost same between participants who were using dietary supplement and medicine (Both) and participants who were using dietary supplement only (DS). In addition, there was no clear difference in the perceptions of dietary supplements in terms of both safety and efficacy depending on whether a person was taking medicine or not.

Among participants who were using medicines, anxiety was almost the same between dietary supplement users (Both) and non-users (PM) for both the present and the future (Table 6). On the other hand, among participants who were not using medicines, anxiety was almost same between dietary supplement users (DS) and non-users (None) at present, but it was higher for dietary supplement users (DS) than non-users (None) in the future. It seems that future health concerns are one of the factors for dietary supplement use, even if respondents are healthy at present. Indeed, some people use dietary supplements for the prevention of diseases. At this time, there are many studies that examine the effect of dietary supplements, such as probiotics [46,47], B-complex multivitamin/mineral supplementation [48], magnesium [49], and omega-3 [50] for anxiety, but there are few reports on whether anxiety towards health encourages people to use dietary supplements. Anxiety was associated with dietary supplement/complementary alternative medicine in cancer survivors [51], women with early breast cancer [52], and patients with liver disease [53] but not healthy subjects.

The HFNet consumer navigation site contains selected information that dietary supplement users should be aware of, along with the consumers intending to use them. However, the awareness of this website is low (0.4–2.2% in this study), regardless of whether people take medicines or use dietary supplements. On the other hand, “Information for consumers related to COVID-19” on HFNet provides information on dietary supplements and materials that claim to prevent coronavirus infection without any scientific grounding. The awareness of this site was about 33% among dietary supplement users [18]. This may be due to the fact that the site was mentioned several times in the media and ranks highly in Japanese Google searches for the keywords “new corona virus” and “dietary supplement”. In addition, pharmacists and dieticians believe that sufficient information on interactions between dietary supplements and drugs is not available, both in Japan [26] and other countries [15]. In this regard, we actively put the information on the interactions between dietary supplements and drugs on HFNet. Even though only 30% of pharmacists and dieticians know this database, most of them use it to obtain information on the interactions between dietary supplements and drugs [26]. In this regard, raising awareness of this database among not only consumers but also healthcare professionals is the first priority issue for the prevention of adverse events of dietary supplement use in patients.

There are both strengths and limitations to this study. An online survey can easily select a target population and randomly allocate participants to each group. Especially during the COVID-19 pandemic, face-to-face and mail surveys are difficult to carry out. In this regard, we should collect sufficient samples, even in older generations. However, participants were limited in terms of the registrants of surveyed companies, and the characteristics of the internet survey participants included a high level of education, a high income, and high socioeconomic status [54]. Therefore, we have to consider this point when making generalizations, especially for older generations. In addition, we did not ask about details of dietary supplements that participants were using. Therefore, we could not estimate the risk of interaction between dietary supplements and prescription medicines in this study. 

## 5. Conclusions

In this survey, many consumers were using dietary supplements as a self-care practice. However, the perception regarding dietary supplements showed that many dietary supplement users considered them safe and effective, and this perception was independent of whether they were taking medicines or not. In addition, many dietary supplement users would potentially use dietary supplements and prescribed medicine concomitantly. In this regard, information about the risk of interaction should be provided, not only to patients taking medicines but also directly or indirectly to dietary supplement users via healthcare professionals.

## Figures and Tables

**Table 1:** Characteristics of respondents.

	*n*	%
Total	10,129	
Sex		
Male	4744	46.8
Female	5385	53.2
Age (years)		
20–29	1917	18.9
30–39	2639	26.1
40–49	2281	22.5
50–59	1696	16.7
60 and older	1596	15.8

**Table 2:** The prevalence of medicine and dietary supplement usage.

	Total	Male	Female	*p*-Value
Medicine				
I am currently taking prescription medicines.	32.6	32.9	32.4	0.019
I am currently taking OTC medicines.	7.5	8.2	6.8	
I do not take any medicine.	59.9	58.9	60.8	
Dietary Supplements				
I am currently using dietary supplements.	38.9	38.9	42.0	<0.001
I am not using but I used to use dietary supplements.	30.5	30.5	33.6	
I have never used dietary supplements.	30.6	30.6	24.5	

*n* = 10,129 (4744 males and 5385 females). Results are shown as percentages (%) of the total or for each sex, respectively. The difference between males and females was examined using the chi-squared (χ^2^) test.

**Table 3:** Concomitant use of dietary supplements and medicines.

	Dietary Supplement Use			
	Currently	Past	Never	*p*-Value
I am currently taking prescription medicines.	14.7	10.2	7.7	<0.001
I am currently taking OTC medicines.	4.7	2.0	0.8	
I do not take any medicine.	19.6	18.3	22.0	

*n* = 10,129 (4744 males and 5385 females). Results are shown as percentages (%) of overall participants. The differences among groups were examined using the chi-squared (χ^2^) test.

**Table 4:** Practical situation of self-care.

	I Am Conducting	I Want to Conduct It in the Future	I Conducted It in the Past, but I Do Not Do It Now	I Am Not Interested/I Am Not Going to Conduct
Regular health check	61.8	21.7	8.9	7.6
Vaccination	58.0	17.1	10.5	14.4
Securing sleep time	56.1	35.1	5.6	3.2
Rest/refresh	53.3	36.2	6.8	3.7
Balanced diet	47.2	38.1	7.1	7.6
Moderate exercise	37.4	35.8	18.5	8.3
Dietary supplement use	36.8	16.3	21.6	25.3
Consult a doctor	31.0	28.0	11.9	29.1
OTC medicine use	27.8	18.0	17.3	36.9
Health application use	19.0	25.9	14.2	40.8
Consult a pharmacist	14.4	29.9	9.6	46.0
Consult a dietitian	2.4	29.0	7.8	60.8

*n* = 10,129 (4744 males and 5385 females). Results are shown as percentages (%) within each practice group.

**Table 5:** Characteristics of respondents.

	Both	DS	PM	None
Total	500	500	500	500
Sex				
Male	250	250	250	250
Female	250	250	250	250
Age (years)				
20–29	100	100	100	100
30–39	100	100	100	100
40–49	100	100	100	100
50–59	100	100	100	100
60 and older	100	100	100	100
Disease (Medication ^1^)				
Hypertension	107 (103)	5 (1)	113 (108)	19 (5)
Diabetes mellitus	48 (42)	1 (0)	46 (41)	4 (2)
Dyslipidemia	104 (81)	29 (8)	76 (68)	14 (5)
Stroke	11 (10)	0 (0)	6 (4)	1 (0)
Heart disease	12 (8)	1 (0)	17 (16)	1 (0)
Cancer	21 (11)	5 (0)	13 (8)	3 (0)
Others	143 (132)	21 (1)	142 (131)	13 (3)

^1^ Medication including both prescription medicine users and OTC medicine users. Both: dietary supplement and prescription medicine users, DS: dietary supplement users, PM: prescription medicine users, and None: non-users.

**Table 6:** Anxiety towards health.

Group	Present	Future
Both	3.42 ± 0.95 ^a^	3.74 ± 0.91 ^a^
DS	2.87 ± 0.98 ^b^	3.39 ± 0.99 ^b^
PM	3.33 ± 1.03 ^a^	3.61 ± 0.97 ^a^
None	2.72 ± 1.00 ^b^	3.06 ± 1.01 ^d^

*n* = 500 in each group. Results are shown as the mean ± SD. The difference among groups was examined using one-way ANOVA with Dunnett’s post hoc tests. Different letters indicate statistically significant differences among groups within Present and Future, respectively. Both: dietary supplement and prescription medicine users, DS: dietary supplement users, PM: prescription medicine users, None: non-users.

**Table 7:** Attitude towards dietary supplement advertisements in the hypothetical setting.

	Medicine Only	Dietary Supplement Only	Use Both	Do Not Use Both	Others	*p*-Value
Both	48.6	2.0	40.8	5.4	3.2	<0.001
DS	42.6	5.8	36.0	13.0	2.6	
PM	68.6	1.4	11.2	17.6	1.2	
None	42.2	1.8	14.2	40.0	1.8	

*n* = 2000. Results are shown as percentages (%) within each consumer group. The difference among groups was examined using the chi-squared (χ^2^) test. Both: dietary supplement and prescription medicine users, DS: dietary supplement users, PM: prescription medicine users, None: non-users.

**Table 8:** Perception of dietary supplements.

	Strongly Disagree	Disagree	Neither Agree Nor Disagree	Agree	Strongly Agree	*p*-Value
It is safe because it is food						
Both	2.0	13.8	36.8	44.4	3.0	<0.001
DS	1.8	11.4	38.8	45.2	2.8	
PM	10.2	24.2	49.4	15.8	0.4	
None	7.8	19.8	51.2	18.8	2.4	
It is safe because it is made from natural ingredients or herbs.						
Both	2.4	14.8	39.4	39.6	3.8	<0.001
DS	3.4	11.8	40.0	41.4	3.4	
PM	11.0	22.6	49.4	16.6	0.4	
None	8.6	19.8	47.6	22.2	1.8	
It has fewer side effects than medicines						
Both	3.2	11.8	36.0	39.8	9.2	<0.001
DS	1.0	12.2	28.0	52.2	6.6	
PM	9.8	20.2	45.0	23.0	2.0	
None	6.2	16.6	45.2	30.4	1.6	
Concomitant use with medicine is safe because it is food						
Both	5.2	18.8	37.8	34.0	4.2	<0.001
DS	6.0	19.0	38.0	33.4	3.6	
PM	16.6	23.4	43.2	15.4	1.4	
None	10.8	20.0	52.0	15.6	1.6	
It can be effective						
Both	3.8	22.0	45.8	26.6	1.8	<0.001
DS	2.4	20.2	47.4	28.2	1.8	
PM	17.8	33.4	39.8	8.4	0.6	
None	10.0	30.6	48.6	9.0	1.8	
I would like to use a product that has a good reputation based on word of mouth						
Both	5.6	15.4	34.8	39.8	4.4	<0.001
DS	4.6	14.8	31.6	43.0	6.0	
PM	19.8	26.8	36.8	15.0	1.6	
None	15.0	21.6	43.0	18.4	2.0	
I would like to use a product recommended by celebrities or experts						
Both	12.0	24.4	40.4	20.4	2.8	<0.001
DS	11.8	22.6	38.8	24.6	2.2	
PM	26.6	29.2	35.8	7.6	0.8	
None	22.8	23.2	40.6	12.0	1.4	
It can prevent diseases						
Both	4.2	18.6	41.0	33.6	2.6	<0.001
DS	5.2	16.4	41.6	34.4	2.4	
PM	17.0	31.2	39.0	12.6	0.2	
None	13.0	25.6	49.0	11.2	1.2	
It can treat diseases						
Both	14.4	34.6	36.8	12.4	1.8	<0.001
DS	16.0	31.0	39.8	12.0	1.2	
PM	26.8	31.4	34.2	7.2	0.4	
None	19.6	28.8	42.6	7.8	1.2	
It can help to improve eating habits						
Both	3.8	12.6	33.4	45.6	4.6	<0.001
DS	4.2	11.0	31.2	50.0	3.6	
PM	11.2	17.0	39.2	29.4	3.2	
None	10.8	19.0	44.2	24.6	1.4	
It is a nutritional supplement for children who like and dislike it						
Both	5.6	14.8	33.4	43.0	3.2	<0.001
DS	5.2	14.4	32.0	45.0	3.4	
PM	16.2	21.4	41.0	19.4	2.0	
None	11.8	20.2	45.0	21.6	1.4	
It is a nutritional supplement for the elderly						
Both	2.2	8.2	25.4	57.6	6.6	<0.001
DS	2.4	6.0	23.4	62.0	6.2	
PM	11.2	17.0	39.2	29.4	3.2	
None	8.4	14.0	44.2	30.2	3.2	
I want to use it for beauty and weight loss						
Both	7.6	18.0	28.0	38.0	8.4	<0.001
DS	8.6	17.8	29.0	36.8	7.8	
PM	24.0	24.4	35.2	14.8	1.6	
None	20.2	21.4	41.6	14.4	2.4	
I would like to use it for muscle building						
Both	11.0	26.6	30.4	26.8	5.2	<0.001
DS	13.0	22.6	34.8	23.8	5.8	
PM	26.6	27.0	33.2	12.2	1.0	
None	26.0	22.2	40.4	9.6	1.8	
It is hard to take every day						
Both	13.6	36.2	21.2	24.8	4.2	<0.001
DS	9.2	34.2	22.4	29.6	4.6	
PM	5.2	14.6	33.4	33.2	13.6	
None	4.6	10.4	39.8	33.6	11.6	
It is expensive						
Both	1.2	7.0	22.4	46.4	23.0	<0.001
DS	0.8	6.8	21.2	50.2	21.0	
PM	1.8	5.6	26.2	33.8	32.6	
None	2.4	4.0	31.8	32.0	29.8	

*n* = 2000. Results are shown as percentages (%) within each consumer group. The difference among groups was examined using the chi-squared (χ^2^) test. Both: dietary supplement and prescription medicine users, DS: dietary supplement users, PM: prescription medicine users, None: non-users.

**Table 9:** Awareness of the Health Food Network consumer navigation site.

	I Know This Site	I Have Seen This Site	I Do Not Know This Site	*p*-Value
Both	2.2	3.8	94.0	0.035
DS	0.6	3.0	96.4	
PM	0.4	1.8	97.8	
None	1.8	3.4	94.8	

*n* = 2000. Results are shown as percentages (%) within each consumer group. The difference among groups was examined using the chi-squared (χ^2^) test. Both: dietary supplement and prescription medicine users, DS: dietary supplement users, PM: prescription medicine users, None: non-users.

**Table 10:** Utilization of the Health Food Network consumer navigation site.

	I Already Use It	I Want to Use It in the Future	I Do Not Want to Use It	I Do Not Need to Use It	I Do Not Know at This Time	*p*-Value
Both	1.2	25.2	8.6	18.8	46.0	<0.001
DS	0.4	20.8	6.4	26.2	45.8	
PM	0.2	7.8	9.8	36.6	45.6	
None	0.4	8.6	14.2	38.2	38.6	

*n* = 2000. Results are shown as percentages (%) within each consumer group. The difference among groups was examined using the chi-squared (χ^2^) test. Both: dietary supplement and prescription medicine users, DS: dietary supplement users, PM: prescription medicine users, None: non-users.

## Data Availability

The data presented in this study are available upon request from the corresponding author.

## Supplementary Materials

The following supporting information can be downloaded at: https://www.mdpi.com/article/10.3390/nu14194114/s1, File S1: Questionnaire.
