# Supplementary material for: Differences in the Perception of Dietary Supplements between Dietary Supplement/Medicine Users and Non-Users
Source: Nutrients. 2022 Oct 3;14(19):4114. doi: 10.3390/nu14194114 (PMC9572052; doi:10.3390/nu14194114)
Supplement: Supplementary file 1 [file nutrients-14-04114-s001.zip › nutrients-1921305-supplementary.pdf]

Questionnaire

Preliminary survey

SC1 Are you currently taking medicines?

1. Yes, I am currently taking prescription medicines.
2. Yes, I am currently taking OTC medicines.
3. No, I do not take any medicine.

SC2 Are you currently using dietary supplements?

1. Yes, I am currently using dietary supplements.
2. No, I am not, but I used to use dietary supplements previously.
3. No, I have never used dietary supplements.

SC3 Do you currently conduct each self-care practice?

	I am practicing.	I want to practice it in the future.	I practiced it in the past, but I do not do it now.	I'm not interested / I'm not going to practice.
Balanced diet				
Moderate exercise				
Rest / refresh				
Securing sleep time				
Dietary supplement use				
OTC medicine use				
Regular health check				
Health application use				
Vaccination				
Consult a doctor				
Consult a pharmacist				
Consult a dietitian				

Further survey

Q1 Have you received a diagnosis of the disease from a doctor?

1. I have never been diagnosed with any disease.
2. Hypertension
3. Diabetes mellitus
4. Dyslipidemia (high cholesterol and triglycerides)
5. Stroke (Infarction, Hemorrhage, Subarachnoid hemorrhage, etc.)
6. Heart disease (myocardial infarction, angina pectoris, etc.)
7. Cancer
8. Others

Q2 How much do you feel anxiety towards your health right now?

5-point Likert-type scales (1 = Not at all to 5 = Extremely anxious)

Q3 How much do you feel anxiety towards your future health?

5-point Likert-type scales (1 = Not at all to 5 = Extremely anxious)

Q4 Your doctor advised that you might have XX (some kind of disease) and you should take medicine for it. On your way home, you saw an advertisement in the train, and it stated that "This dietary supplement improves XX". What would you do?

1. I will use only medicine (including continuation of medical care).
2. I will use only dietary supplement.
3. I will use both medicine and dietary supplement.
4. I will not use any of them.
5. Others.

Q5 How do you think about dietary supplement?

	Strongly disagree	Disagree	Neither agree nor disagree	Agree	Strongly agree
It is safe because it is food					
It is safe because it is made from natural ingredients or herbs					
It has fewer side effects than medicines					
Concomitant use with medicine is safe because it is food					
It can be effective					
I would like to use a product that has a good reputation based on word of mouth					
I would like to use a product recommended by celebrities or experts					
It can prevent diseases					
It can treat diseases					
It can help to improve eating habits					
It is a nutritional supplement for children who like and dislike it					
It is a nutritional supplement for the elderly					
I would like to use it for beauty and weight loss					
I would like to it for muscle building					
It is hard to take every day					
It is expensive					

Q6 Have you ever seen “Health Food Network”?

(View https://hfnet.nibion.go.jp/)

1. No, I have never seen this site.
2. Yes, I have ever seen this site.
3. Yes, I know this site, but I do not use it.
4. Yes, I know this site, and I am using it.

Q7 Have you ever seen “Health Food Network consumer navigation site”?
(View https://hfnet.nibiohn.go.jp/notes/detail.php?no=2311)

1. No, I have never seen this site.
2. Yes, I have ever seen this site.
3. Yes, I know this site well.

Q8 Do you want to use “Health Food Network consumer navigation site”?

1. I am already using this website.
2. I would like to use this site in the future.
3. I do not want to use this site.
4. I do not need to use this site.
5. I do not know at this time.

Thank you for your cooperation
